# Supplementary material for: The Arabidopsis LHT1 Amino Acid Transporter Contributes to Pseudomonas simiae-Mediated Plant Growth Promotion by Modulating Bacterial Metabolism in the Rhizosphere
Source: Plants (Basel). 2023 Jan 12;12(2):371. doi: 10.3390/plants12020371 (PMC9867026; doi:10.3390/plants12020371)
Supplement: Supplementary file 1 [file plants-12-00371-s001.zip › plants-2132899-supplementary.pdf]

plants

Supplementary Figures

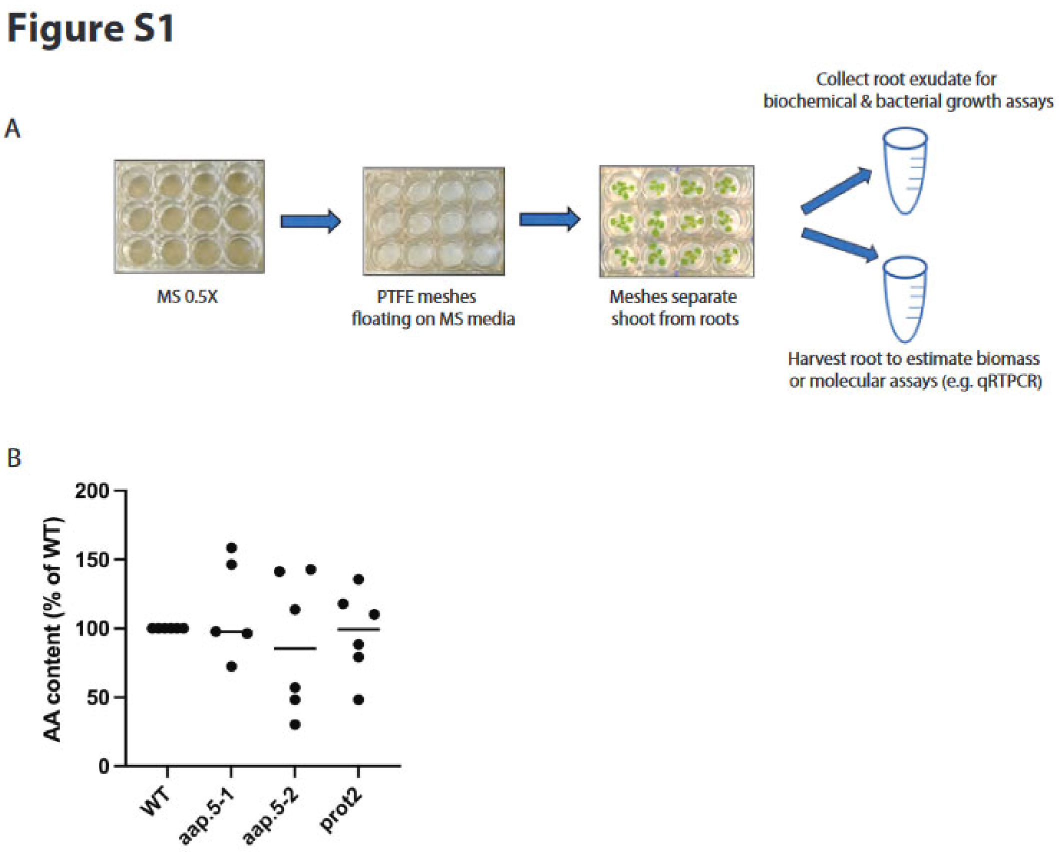

Figure S1. (**A**) Workflow to obtain root exudates. Twelve-well plates were loaded with 1 mL of 1x MS buffer supplemented with 0.5% sucrose and 0.5 g/L MES. Seeds were sown on sterile polytetrafluoroethylene (PTFE) meshes and placed floating on top of the medium. After 12 days, the medium was replaced with 0.5x MS liquid medium without sucrose, and plants were allowed to grow for three additional days. Importantly, in this condition, roots grow submerged in the medium while shoots remain in the air space of the well. Root exudates were then collected and filter-sterilized for further processing or testing. (**B**) Total AA content in *aap5* and *prot2* root exudates is similar to that of WT plants. Seeds of sequence-verified homozygous mutants were sown, and exudates were obtained, as described in (**A**). Colorimetric quantification of AAs was carried out as described in Methods. Each dot corresponds to a biological replicate and is the median AA content per mg of roots of 5 seedlings per genotype relative to the wild type (as a percentage). Experiments were repeated 6 independent times. One-way ANOVA followed by Tukey's post hoc test shows no statistically significant differences between WT and the mutant genotypes.

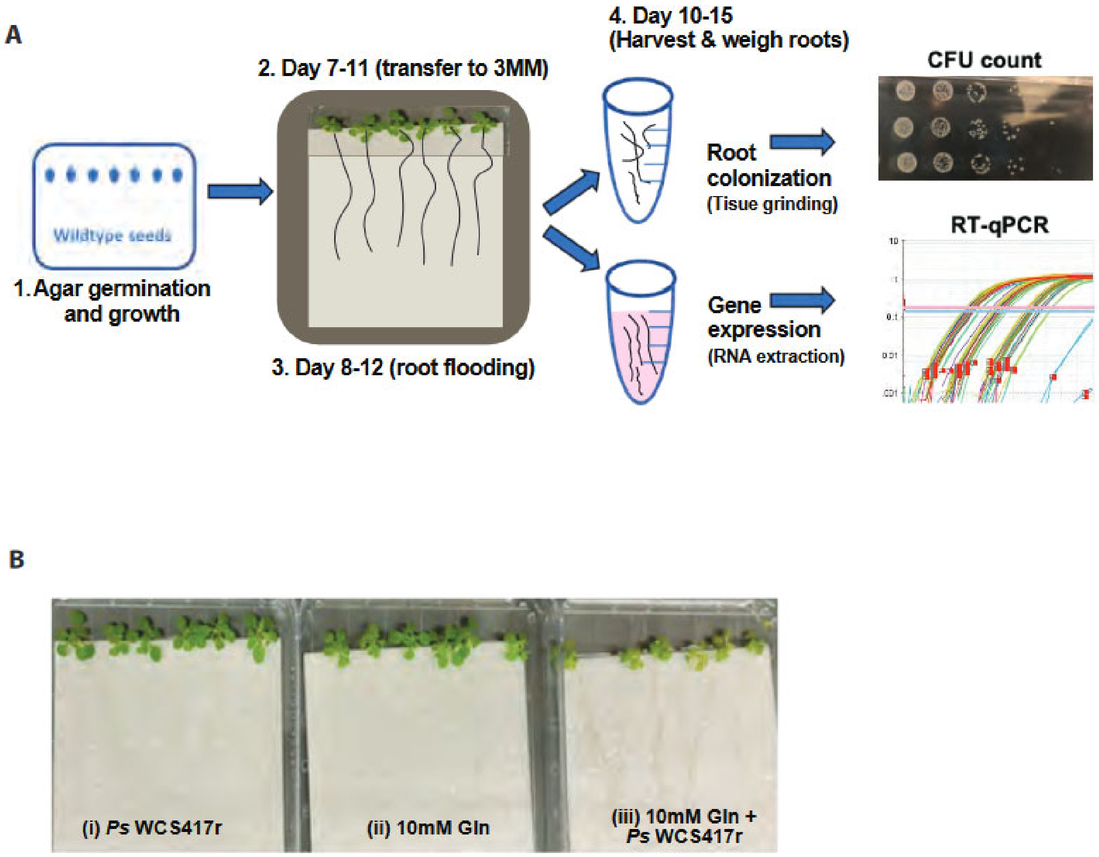

Figure S2. (**A**) Workflow to assess colonization and gene expression in roots. 1. Stratified seeds are germinated in 1x MS agar with 0.5% sucrose. 2. At days 7 to 11, uniformly growing seedlings were transferred to square plates containing autoclaved 3 MM paper wetted with 0.5x MS without sucrose (composed image to reveal roots position and size). Plates were sealed and incubated horizontally for 1 day to allow roots to attach to the 3 MM paper. 3. At days 8 and 12, the bottom of the 3 MM paper is flooded with a suspension of *Ps* WCS417r (final OD_{600nm} = 0.2) or sterile 0.5x MS as a no-growth (contamination) control. The bacteria are resuspended in exudates obtained from WT or *lht1*, and WT exudates were supplemented with Aas, as indicated. 4. After 24–72 h of exposure to exudates ± *Ps* WCS471r ± AAs, roots are harvested, weighed, and processed to obtain RNA or to count bacterial colony-forming units (CFU). (**B**) Representative pictures of seedlings in square plates on 3 MM paper substrate. At day 13, roots were flood-treated with: (i) *Ps* WCS417r OD_{600nm} =0.2 (control), (ii) 10 mM Gln, or (iii) *Ps* WCS417r and Gln at final OD_{600nm}=0.2. The images show symptoms 3 days after treatment.

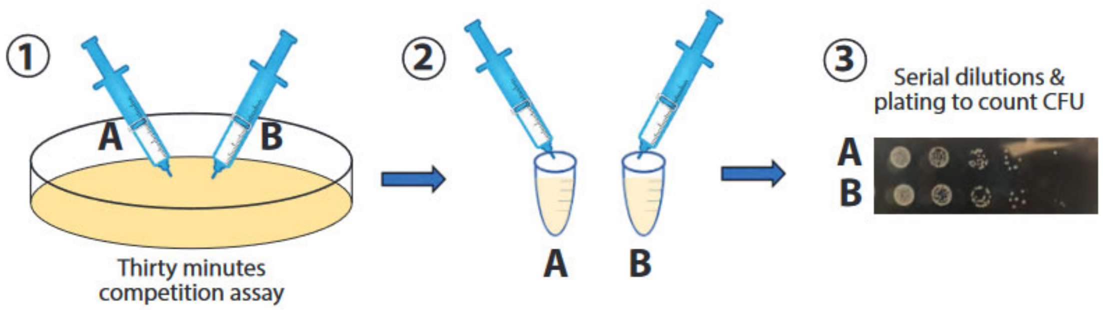

Figure S3. Workflow of competitive chemotaxis assay. (**1**) A Petri dish is filled with 40 mL of *Ps* WCS417r freshly resuspended chemotaxis buffer at a final OD_{600nm} = 0.002. Filter-sterilized root

exudates (200 μL) or fresh MS medium (negative control) were loaded into 1 mL sterile syringes (without the needles). The tips of the two syringes to be tested in the competitive assay were then immersed just under the surface of the bacterial suspension contained in the Petri dish. (**2**) After 30 min, the content of each syringe was transferred to independent 1.5 mL tubes. (**3**) Serial dilutions were plated on LB-agar plates to count colony-forming units (CFU).

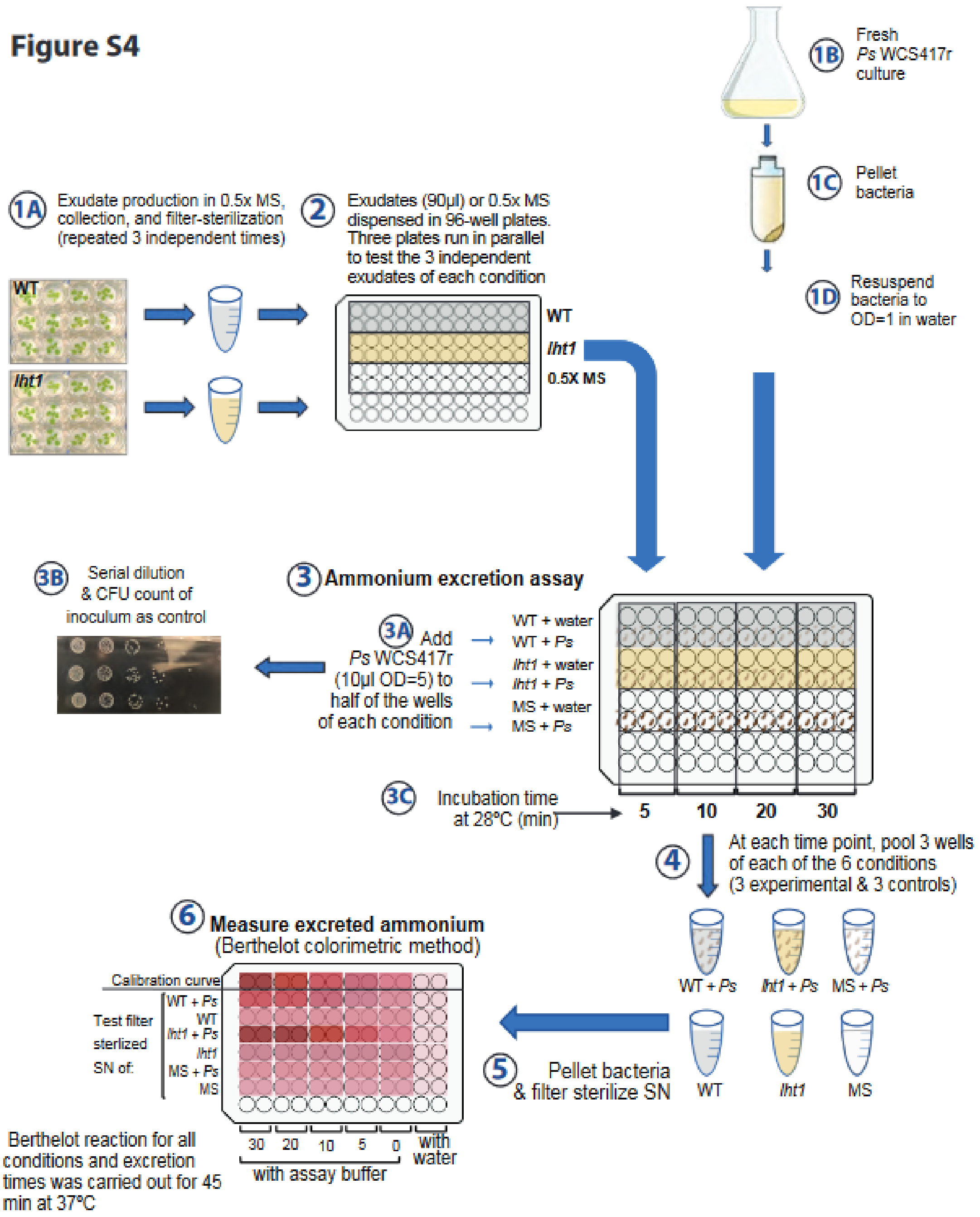

Figure S4. Workflow of *Ps* WCS417r excreted-ammonium assay. WT and *lht1* exudates were collected (**1A**), as described in Methods. Aliquots of 90 μL of each exudate were transferred to 24 wells of a 96-well plate (**2**). Whereas 12 wells of each exudate were inoculated with freshly harvested (**1A**) and three times washed (**1B**) *Ps* WCS417r to reach a final $OD_{600nm} = 0.02$ (**1C**), the remaining 12 wells of each condition were left bacteria-free to be used as negative controls. Plates were incubated at 28 °C with constant shaking (**3**). At 5, 10, 20, and 30 min, 3 wells of each experimental and 3 wells of each control condition were harvested (**3A**). In parallel, 10 μL aliquots were used to assess CFUs (**3B**). After centrifugation for 5 min at 10,000± *g*, the 3 supernatants from each treatment and control

condition were pooled (**4**) and filtered-sterilized (**5**). Aliquots of the supernatants were used to quantify ammonia (**6**) using the ammonia assay kit from AbCam (Cat # ab102509), according to the manufacturer's guidelines. The experiment was repeated 3 times with independent batches of fresh exudates.
